# Shaking Up Photochemistry: The Future Frontiers of Mechanophotocatalysis

**DOI:** 10.1021/acscentsci.5c01770

**Published:** 2026-01-01

**Authors:** Francis Millward, Eli Zysman-Colman

**Affiliations:** Organic Semiconductor Centre, EaStCHEM School of Chemistry, 7486 University of St Andrews, St Andrews KY16 9ST, U.K.

## Abstract

Solution-state photocatalysis is fundamentally reliant on the use of organic solvents, which are associated with significant safety, sustainability, and implementation challenges for conducting light-driven reactions. Mechanophotocatalysis tantalizingly addresses these issues by significantly reducing the use of reaction solvents, using mechanical mixing to mediate light-driven transformations. In this Outlook, we examine the motivations for combining photocatalysis with mechanochemistry, assess how this nascent methodology has evolved, and speculate on future research directions that should be explored in order for mechanophotocatalysis to emerge as a useful and complementary methodology for conducting photochemical reactions under solvent-minimized conditions.

## Graphical abstract

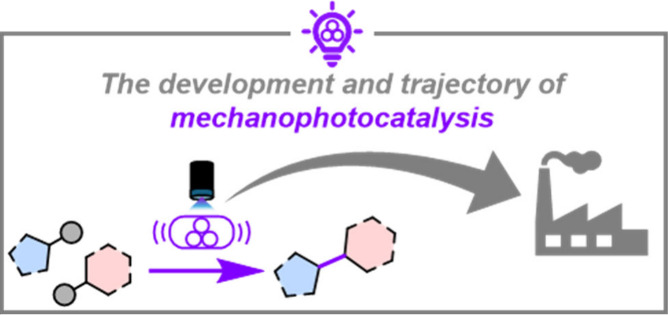

## Photocatalysis: The Solvent Problem

Photocatalysis, and photochemistry more generally, is a powerful tool for constructing complex molecules from abundant coupling partners in a small number of synthetic steps using light to drive reactions (Figure 1a).^1–7^ In these transformations, organic solvents are employed to solubilize the reaction components, while stirring facilitates efficient mixing of the system. However, this near universal reliance on organic solvents introduces significant sustainability, safety and workflow issues (Figure 1b). Many commonly employed solvents in photocatalysis reactions are toxic and/or particularly flammable organic liquids, including *N,N*-dimethyl­acet­amide (DMA), *N,N*-dimethylformamide (DMF), acetonitrile (MeCN) and dimethyl sulfoxide (DMSO).^8–12^ Despite their apparently simple role as reaction media, solvents represents the greatest contributor to the total mass in a typical reaction system and become the largest source of waste throughout a multistep route.^12–15^ Solvents may also play unpredictable roles in reaction mechanisms, mandating optimizing the solvent identity and concentration, which can be time-consuming. The explorable chemical space is often restricted to components (reagents, additives, and catalysts) that are soluble in that solvent. The removal of high-boiling-point solvents like DMA and DMSO is energy-intensive and can hinder high-throughput experimentation (HTE) workflows. Furthermore, solution-state photochemical reactions are difficult to scale in batch, mainly due to inefficient light penetration in larger vessels as a result of the Beer–Lambert law, which describes the exponential attenuation of light intensity through a reaction mixture as a function of increasing path length and concentration of the light-absorbing materials. As a result, photochemical reactions are often conducted as dilute solutions, which increases solvent waste and leads to nonuniform irradiation and slower reaction kinetics within batch reactors.^16^ While the use of continuous flow reactors addresses this issue by restricting the path length, these systems still rely on organic solvents, and often struggle with insoluble substrates without the incorporation of specialized technologies.^16,17^ Solution-state photocatalysis reactions can also be sensitive to aerobic conditions, as oxygen dissolved in solution can act as a competitive quencher of the excited state of the photocatalyst. While this can be desirable in systems using oxygen as a ‘green’ oxidant,^18,19^ many reactions require rigorous degassing, which increases the complexity and cost of setting up reactions. However, many of these issues could, in principle, be addressed using mechanochemistry in combination with light excitation.

**Figure 1:**
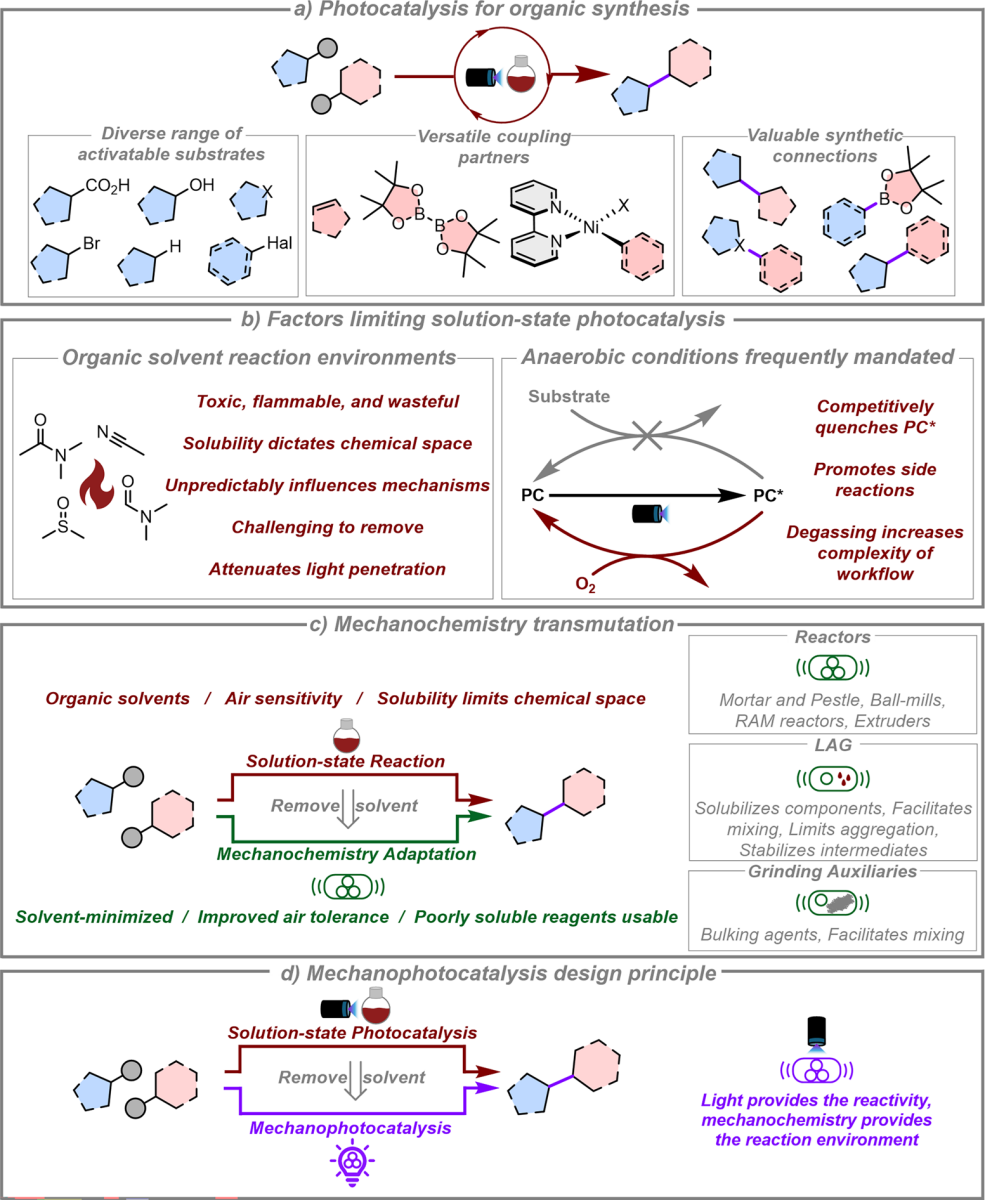
a) Demonstrating the value of photocatalysis for organic synthesis by highlighting the diverse range of activatable substrates that can be used for valuable transformations. For examples, see refs 49–54 . b) Solvent-induced issues for photocatalysis. PC = photocatalyst. c) Mechanochemistry transmutations of conventional reactions and the potential associated benefits, along with the additional tools that can be used, including reactor examples, LAG (liquid assisted grinding) and grinding auxiliaries. d) The mechanophotocatalysis design principle; taking an existing solution-state photocatalysis reaction, removing the bulk solvent, and using mechanochemistry tools to facilitate mixing.

Despite their apparently simple role as reaction media, solvents represent the greatest contributor to the total mass in a typical reaction system and become the largest source of waste throughout a multistep route.

## Mechanophotocatalysis: The Design Principle

Mechanochemistry has emerged as a versatile tool for organic synthesis principally due to its ability to mediate solvent-minimized versions of known solution-state reactions (Figure 1c).^20–22^ Here, bulk solvent is removed from the reaction, and mechanical grinding or milling mixes the components and continuously generates new reactive surfaces.^23–25^ Notable examples of this approach include solvent-minimized S_N_Ar reactions,^26,27^ amide couplings,^28,29^ Wittig olefinations,^30^ palladium and nickel-catalyzed cross-couplings,^31–37^ and transformations using reactive zerovalent metals.^38,39^ While many reactor technologies exist, from the mortar and pestle to twin-screw extruders and resonant acoustic mixing (RAM) reactors, mechanochemistry on a laboratory scale is most commonly conducted using ball mills.^25^ Neat reactions can be milled; however, inert solid grinding auxiliaries and liquid-assisted grinding (LAG) agents can also be added to increase reaction efficiencies.^25,33,40^ Aside from reducing waste generation associated with reaction solvent,^41^ mechanochemical reactions can be faster,^27,42^ can facilitate access to new chemical species and reaction selectivities,^43,44^ and be more tolerant to aerobic conditions^39,45^ and poorly soluble reaction components^42,46–48^ than their solution-state analogues, enabling simplified workflows and access to new chemical space.

Considering the solvent-associated issues for conducting photocatalysis reactions, and the apparent benefits of adapting reactions to a mechanochemical environment, it is apparent that the successful merger of these two complementary methodologies could lead to a paradigm shift in how light-driven reactions are conducted (Figure 1d). Using this *mechanophotocatalysis* approach, reactions would be driven using light (and often a photocatalyst), while mechanical grinding is responsible for mixing the reaction, and generating new surfaces that can be irradiated by light. A small number of reports exist that document the use of different mechanochemistry tools for mediating photochemical reactions in the absence of bulk solvent. We refer to this field as *mechanophotocatalysis*, as this term encapsulates a chemistry that is mediated by light, while the mechanochemistry is responsible for mixing,^55–57^ while others have used the terms *solid-state photochemistry*,^58^ *mechanochemically assisted solid-state photocatalysis*,^59^ or *photomechanochemisty*.^60–62^ A recent review documents literature examples up to the end of 2024;^61^ however, since then, there have been several important advances in the field, including more industrially relevant reactions being adapted to solvent-minimized conditions, and RAM being used to mediate these processes. To facilitate the continuing acceleration of research in this exciting new field, we summarize key advances for applications in organic synthesis (Figures 2, 3, and 4), providing context for a discussion of how we anticipate this rapidly evolving methodology will develop in the future.

## Mechanophotocatalysis: Development and Trajectory

Examples of solvent-free photochemistry reactions, conducted in the absence of mechanical agitation, have classically been restricted to simple homodimerization and cycloaddition reactions of crystalline solids.^63–67^ In the context of a more complex mixture of reactants, Yu, Wang, and co-workers reported the formation of quinolines from 2-vinylanilines and sulfoxonium ylides via the pregrinding of the solid reaction mixture before irradiation with a blue LED with simultaneous heating.^68^ Silica-supported photocatalysis reactions on TLC plates have also been demonstrated.^69^ A key issue limiting the generalizability and scalability of these static irradiation approaches is the nonuniform irradiation of the whole reaction mixture; in the absence of continuous mixing, only the surface of a solid reaction mixture will be irradiated. Furthermore, without efficient mass transfer, reactions are likely to be sluggish, as solid-state reactions are limited to the interface layers between the reactants.^23^ However, continuous agitation that generates new surface areas using specialized mechanical mixing tools, such as ball mills, should address both these issues. Therefore, the most effective approach for developing a generalizable protocol for solvent-minimized photocatalysis reactions likely involves simultaneous mechanical agitation and light irradiation.

It has already been demonstrated that mechanochemistry can be readily combined with other energy sources,^70^ but from a practical perspective the irradiation of reactions in a mechanochemistry reactor requires the use of transparent reaction vessels that are robust enough to withstand mechanical forces, and are chemically resistant. Most commercially available milling jars used in ball mills are made from opaque materials like stainless steel, zirconium oxide, and polytetrafluoroethylene (PTFE). While transparent milling jars made from poly­(methyl methacrylate) (PMMA) are used in mechanochemistry applications, particularly for *in situ* reaction monitoring,^71^ they frequently lack the required chemical resistivity to be useful for the majority of photocatalysis reactions, and can become scratched and opaque over time, leading to poor light penetration.^56^ On the other hand, solution-state photocatalysis reactions are typically conducted using glassware that is often incompatible with high-energy milling. The design of novel reactor and reaction vessel technologies is therefore essential for enabling effective mechanophotocatalysis.

Considering the solvent-associated issues for conducting photo­catalysis reactions, and the apparent benefits of adapting reactions to a mechanochemical environment, it is apparent that the successful merger of these two complementary methodol­ogies could lead to a paradigm shift in how light-driven reactions are conducted.

In 2016, Obst and König reported the solvent-free aerobic oxidation of benzylic alcohols to their corresponding carbonyl analogues (6 examples, 37–74% yield in 24 h) using a low energy rod mill reactor and an organic photocatalyst, riboflavin tetraacetate (RFTA).^72^ The rod mill used a slowly rotating glass rod within a glass reaction tube that generates a thin film of the neat reaction mixture that could be irradiated by an LED. The reaction also proceeded efficiently in the absence of continuous mixing, but heat was found to be essential, as cooling of the reactor led to only trace product formation, suggesting that melting of the reaction mixture was key for intimate mixing of the components in this low energy reactor. König et al. also reported the neat dehalogen­ative coupling of electron-poor aryl halides with pyrroles and phosphites using a similar thin-film approach, this time using a horizontally rotating glass vial with rhodamine 6G (R6G) as a photocatalyst (16 examples, 37–78% yield in 24 h).^73^ As no mechanical grinding force was applied, this system likely relies on the liquid pyrroles and tertiary amine electron-donor to act as solubilizing/dispersion agents, and additional mechanical input would likely be needed for more heterogeneous reactions. While these initial reports relied on lower-energy reactor designs to mediate reactions, the field has predominantly transitioned to using ball mills as the primary mixing and grinding technology.

In 2017, Štrukil and Sajko reported a solvent-free aerobic oxidation of diphenylacetylene to benzil (43% yield in 6 h), using a ball mill combined with glass milling jars, a Teflon milling ball, Eosin Y as a photocatalyst, and sodium sulfate as a grinding auxiliary.^59^ As a reactor, the authors used a commercially available ball mill and incorporated an LED light array placed in close proximity the jar holder positions. In the same year, Hernández reported the solvent-free borylation of aryldiazonium salts in a ball mill using PMMA jars as the reaction vessel (5 examples, 41–68% yield in 45–120 min).^74^ In this instance, irradiation was achieved via wrapping LED light strips around the jars, and Eosin Y was used as a photocatalyst.

**Figure 2:**
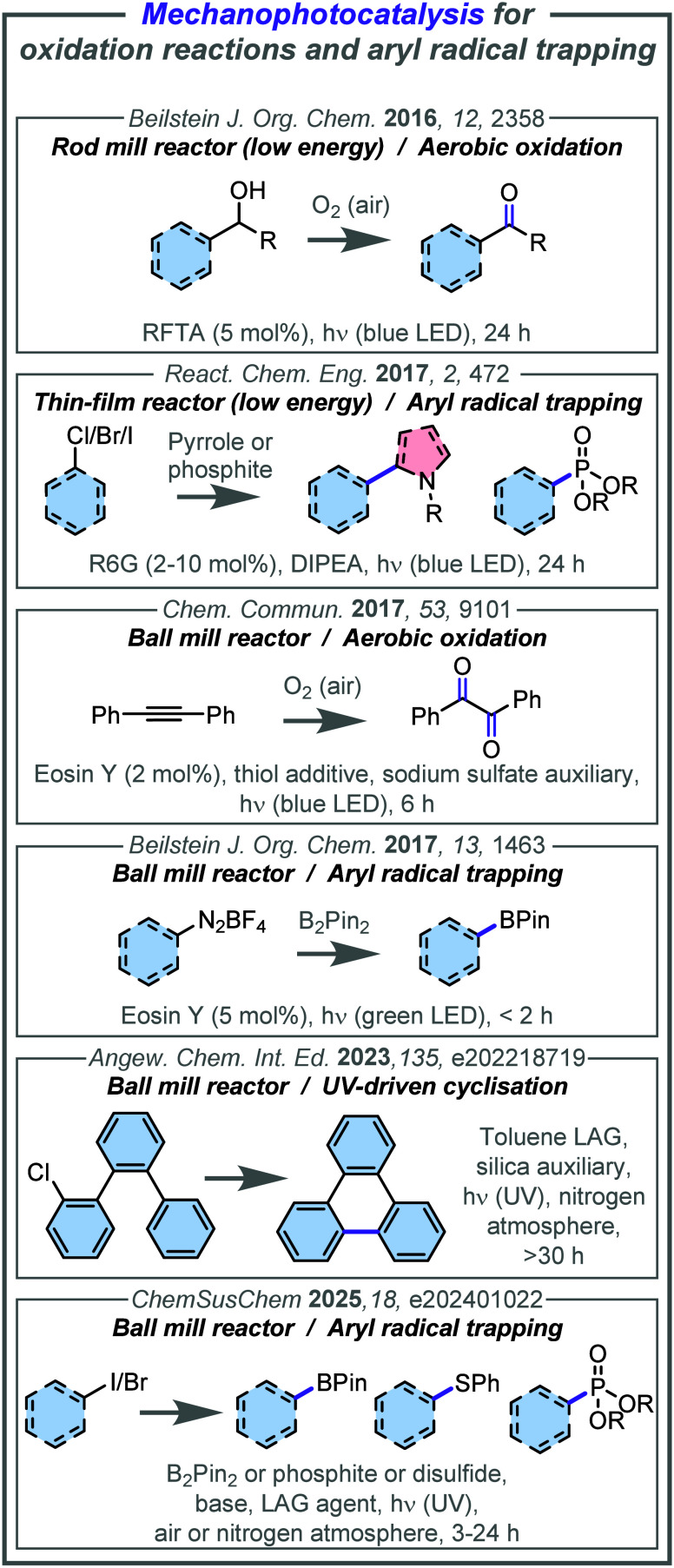
Examples of mechanophotocatalysis for oxidation reactions and aryl radical trapping. Abbreviations: RTFA = riboflavin tetraacetate, R6G = rhodamine 6G, DIPEA = *N*,*N*-diisopropylethylamine.

The UV-light-promoted [2+2] cycloaddition of acenaphthylene (96% in 20 h) has been demonstrated using a ball mill with an LED positioned next to the reaction vessel.^75^ Similarly, vortex mixing has been combined with UV-light irradiation for simple solid-state dimerization reactions.^76,77^ In 2023, Borchardt and co-workers used a UV-light array, a ball mill, and quartz glass milling vessels for the synthesis of polycyclic aromatic compounds (7 examples, 18–92% yield in 30 h or more under a nitrogen atmosphere).^78^ Two years later, the same group expanded upon their previous work by demonstrating the UV-driven dehalogenation of aryl iodides and bromides with subsequent trapping by bis­(pinacolato)­diboron, triethyl phosphite, and disulfides to facilitate solvent-minimized C­(sp^2^)–B, C­(sp^2^)–P, and C­(sp^2^)–S bond formations (22 examples, 11–99% yield in 3–24 h).^79^ Notably, the use of acetonitrile as a LAG agent was found to be crucial for enabling efficient reactivity, and while all reactions could be conducted under air, the borylation yields were higher under an inert atmosphere. An array of mercury lamps were placed near to the jar holder positions. Halasz and co-workers recently reported the *in situ* monitoring of the photoisomerization of azobenzene and the photolysis of adamantane diazirine in a ball mill using Raman spectroscopy.^60^ Kulcsár and co-workers have subsequently used *in situ* Raman monitoring during a [2+2] cycloaddition of *trans*-1,2-bis­(4-pyridyl)­ethene in a ball mill.^80^ Also in 2025, Bantreil and co-workers developed a solvent-minimized protocol for the reductive dehalogenation of α-halo ketones (8 examples, 64–88% yield in 3.5 h), using a ball mill and an epoxy resin reaction vessel.^81^ These examples clearly demonstrate that photochemical reactions can take place in the absence of bulk solvent; however, many of these systems required long reaction times, with limited comparisons to analogous solution-state systems to permit accurate benchmarking of this new methodology. Furthermore, nearly all of these milling approaches necessitated the removal of their mill’s safety shield to place the light sources close to the jar holder positions, leading to potential safety hazards associated with high light leakages and damage to the lights in the event of mechanical failure.

The most effective approach for developing a generalizable protocol for solvent-minimized photo­catalysis reactions likely involves simultaneous mechanical agitation and light irradiation.

In 2024, we demonstrated that mechanophotocatalysis seems to be a generalizable methodology for conducting solvent-minimized photocatalysis reactions using a ball mill equipped with a metal safety shield with a hole above each reaction position that facilitates irradiation from a safe distance (8–10 cm) using *Kessil* LEDs.^56^ This approach limits light leakage from the reactor and provides suitable protection to the researcher. We systematically compared the performance of four mechanistically distinct reactions under both solution-state and mechanophotocatalysis conditions using the same light source. Of particular note was the atom transfer radical addition (ATRA) of sulfonyl chlorides to styrenes, where the aerobic mechanophotocatalysis protocol provided consistently higher yields (70–94%) than the anaerobic solution-state comparison (8–95%) across 8 different substrates in the same 2-h reaction time. Furthermore, the solution-state reaction under air did not proceed; thus, the mechanophotocatalysis protocol provided a dramatically improved tolerance to aerobic conditions in this system. We also reported the pinacol couplings of an aldehyde and a ketone, decarboxylative alkylations of an alkene, and the [2+2] cycloaddition of *trans*-chalcone, all proceeding under solvent-minimized conditions in short reaction times (47–93% in 2–3 h across these three reactions).^56^ In 2025, using the same reactor, we unveiled an open-source mechanophotocatalysis reaction vessel design that reduces the barrier to adoption and provides an opportunity for standardizing the tools used for mechanophotocatalysis reactions.^55^ The metal holder can support multiple 2 mL Eppendorf vials as simple reaction vessels for small-scale reaction screening in a ball mill. We used this approach to conduct solvent-minimized metallaphotoredox reactions, including aryl aminations, and C­(sp^2^)–C­(sp^3^) cross-couplings between aryl halides and alkyl carboxylic acids, bromides, and a trifluoroborate salt (13–86% yield from 26 substrates across 4 reaction classes).^55^ Low loadings of DMA or 1,2-dimethoxyethane as LAG agents were found to be essential for enabling efficient yields in these reactions. Notably, the cross-electrophile coupling reaction possessed an enhanced tolerance to air under the mechanophotocatalysis conditions relative to the solution-state comparison, thereby enabling a 12–52% increase in yield for primary and secondary alkyl bromide coupling partners over the same reaction time. Unfortunately, this approach has thus far been limited to small-scale reaction screening (∼0.3 mmol scale) due to the limited size of the reaction vessels, and new, larger reaction vessels will need to be designed for preparative applications. In an effort to understand the origin of the enhanced tolerance to air that the ATRA and cross-electrophile coupling reactions experienced, we investigated the excited-state quenching behavior of five different photocatalysts by air in the presence and absence of solvent.^55^ We observed more significant quenching of the excited states of the photocatalysts by oxygen in solution than in the solid state, which likely contributes to the enhanced tolerance to aerobic conditions these reactions experienced.

A key strength of mechanochemistry is the ability to mediate reactions with poorly soluble components more efficiently than their solution-state counterparts, as a function of the solvent-minimized grinding environment that facilitates mixing and *in situ* particle size reduction to generate new reactive surfaces.^42,47^ Inspired by this, our group recently used a mechanophotocatalysis platform to replace traditional solution-state sacrificial reductants, such as *N*,*N*-diisopropylethylamine (DIPEA) and the Hantzsch ester, with sodium ascorbate as a safer, more sustainable, and easier to remove alternative in a photocatalysed pinacol coupling reaction (11 examples, 20–75% yield in 4 h).^57^

**Figure 3:**
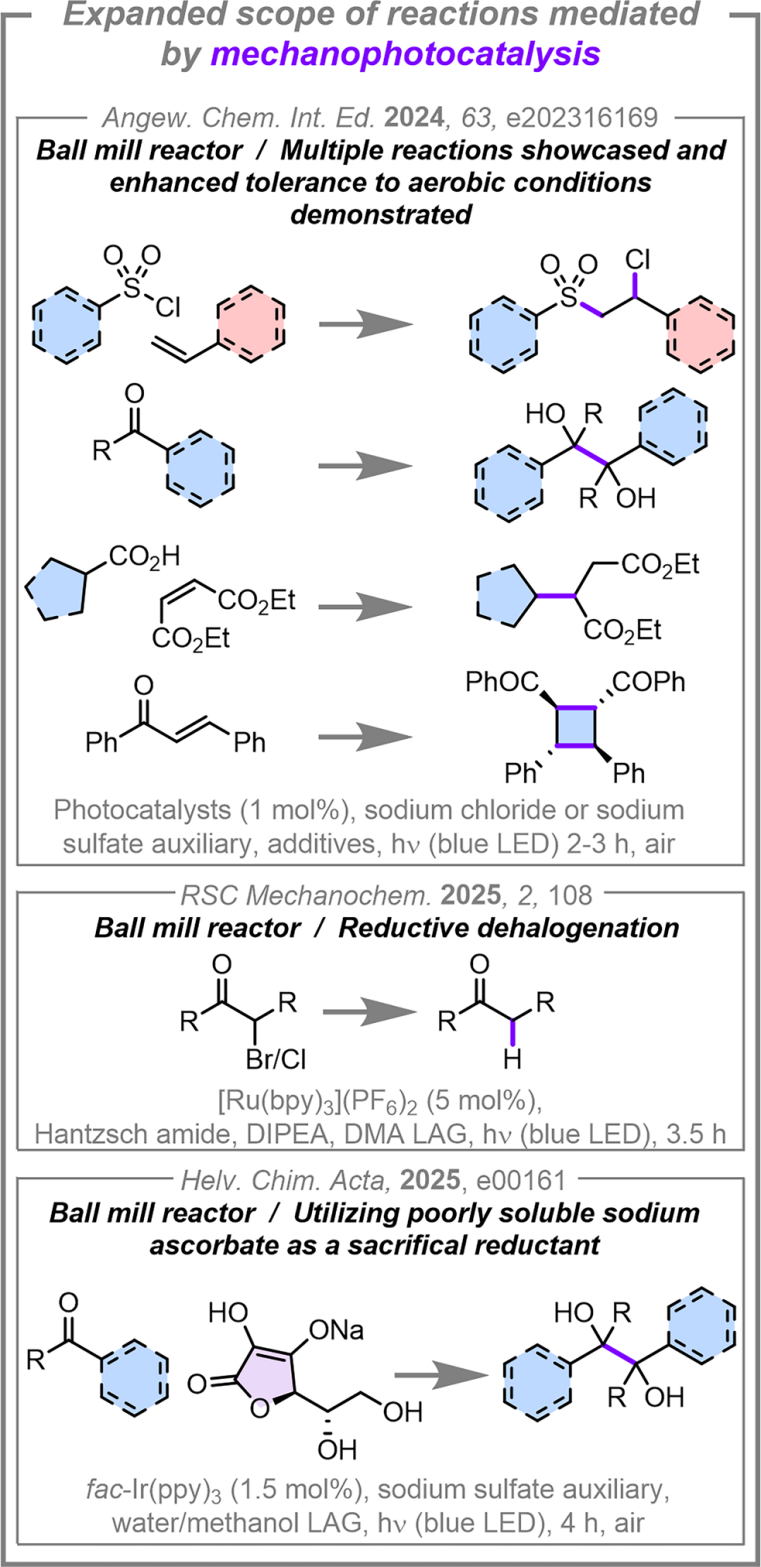
Expanded scope of mechanophotocatalysis reactions. Abbreviations: DIPEA = *N*,*N*-diisopropylethylamine, DMA = *N*,*N*-dimethylacetamide.

While manual grinding typically leads to more inconsistent results than when using automated ball mills for mechanochemistry reactions, this approach can still be used for solvent-minimized photocatalysis reactions. As a recent example, Peng, Xie, and co-workers reported the rapid synthesis of vinyl sulfones from sulfinic acids and alkenyl sulfonium salts using sunlight as the light source (39 examples, 62–92% yield in under 10 min).^82^ An alternative approach to enabling photochemistry in the solid-state was reported by Wu, Wang and co-workers, who used mechanoluminescent materials (which generate light upon mechanochemical stimulation) as the photon source inside conventional mechanochemistry vessels to drive Hofmann–Löffler–Freytag-type cyclizations (21 examples, 39–93% yield in 2 h) and for the synthesis of vinyl sulfones via an electron donor–acceptor (EDA) complex activation (19 examples, 39–93% yield in 3 h).^83^ Curiously, the EDA reaction performed poorly when conducted under air. This approach could be a useful tool for reactions that can be driven by lower energy light (>500 nm), as it does not require any specialized reactor technologies aside from a ball mill; however, it remains to be seen if this alternative approach can be used to drive reactions requiring higher energy (blue) light or a high photon flux.

Recently, RAM has been used to drive solvent-minimized photochemical reactions. In contrast to ball milling, which uses milling balls to grind and mix reactions, RAM induces mechanical mixing by the vertical oscillation of the reaction vessel at a set frequency (typically 60 Hz), with the energy of the system being modulated by varying the amplitude of the oscillation.^84,85^ The induced acoustic energy enables the rapid mixing of the reaction contents in the complete absence of milling media, meaning glass reaction vessels can be used, and the reaction setup is simplified. Consequently, this ability to use standard laboratory glassware as reaction vessels in a mechanophotocatalysis setting obviates the need for bespoke reaction vessels. In 2025, Borchardt, Grätz, and co-workers translated their previously developed UV-promoted dehalo­gen­ative cyclization and phosphonation of an aryl halide to a RAM reactor, enabling the scaling of these reactions in batch (up to 60% yield on a 3.8 mmol scale for the phosphonation reaction in 3 h, and up to 53% on a 3.97 mmol scale for the dehalogenative cyclization in 18 h).^58^ In the same year, Rueping and co-workers conducted solvent-minimized metallaphotoredox catalysis reactions using a RAM reactor equipped with an array of four *Kessil* LEDs.^62^ C­(sp^2^)–heteroatom bond formations between aryl bromides or chlorides and nucleophiles, including aniline derivatives, primary and secondary alkyl amines, alcohols, thiols, and primary sulfonamides, were mediated in short times (>70 total examples, 29–95% yield in <90 min). Notably, loadings as low as 0.01 mol% of the organic photocatalyst 1,2,3,5-tetrakis­(carbazol-9-yl)-4,6-di­cyano­benzene (**4CzIPN**) were tolerated, illustrating the exceptional mixing that can be achieved despite the absence of bulk solvent. Furthermore, the reaction performed more efficiently in the RAM platform in the absence of grinding auxiliaries. This is potentially an advantage of a RAM approach over ball milling mechanophotocatalysis, as ball milling systems have frequently needed to optimize the reaction rheology using these additives.^55,56^ Another advantage of using RAM is the ability to conduct small-scale test reactions and large-scale batch procedures in the same reactor, which is not practical for ball milling reactors. Rueping and co-workers demonstrated the use of 12 × 4 mL vials for small-scale reaction screening, and could scale reactions up to 300 mmol within a larger vessel. Curiously, however, an argon atmosphere was required throughout this work, while our solvent-minimized amination system proceeded efficiently under air.^55^ Rueping and co-workers extended the scope of reactions in their study, showcasing a visible-light promoted copper catalyzed Ullmann C–N cross-coupling, a decarboxylative arylation, an α-amino C­(sp^3^) phosphorylation, the [2+2] dimerization of chalcone, and a decarboxylative EDA system, all achieving high yields of 61–76% on 0.4–1 mmol scales in 90 min.^62^

**Figure 4:**
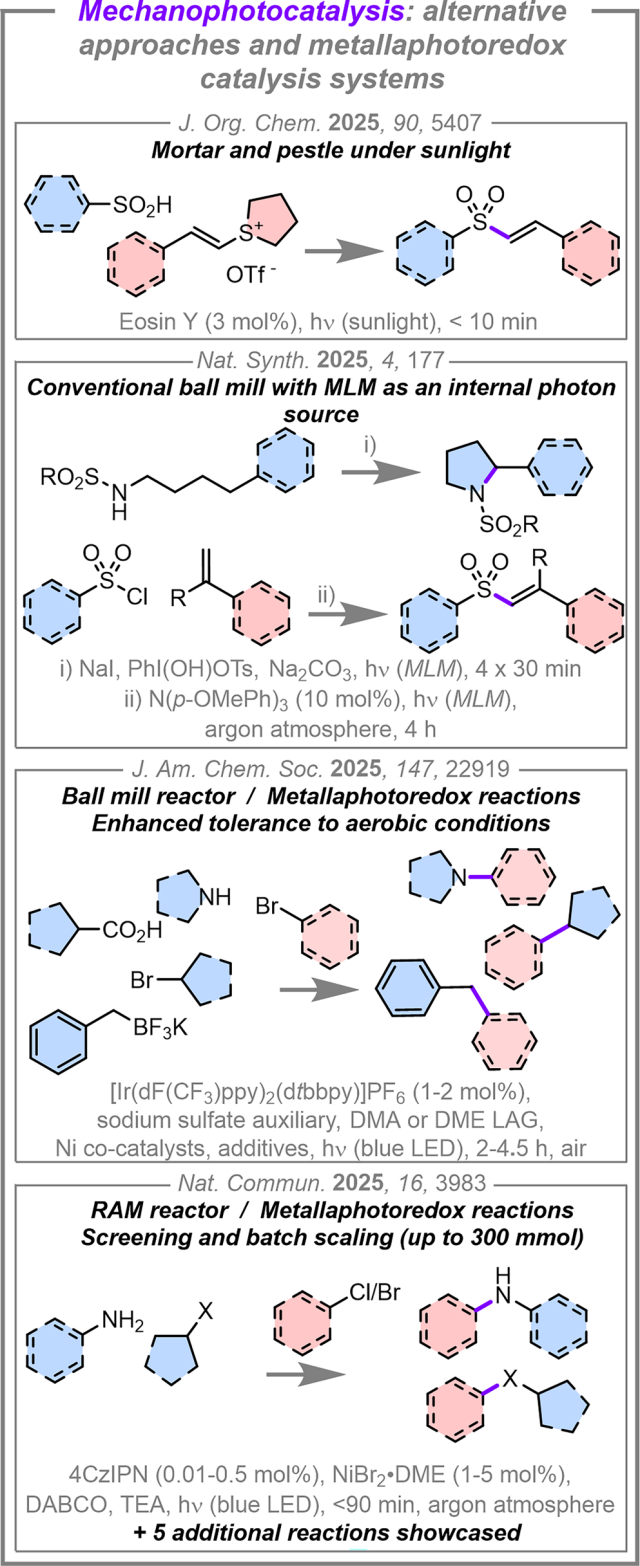
Alternative approaches for mechanophotocatalysis reactions, and examples of solvent-minimized metallaphotoredox catalysis systems. Abbreviations: DMA = *N*,*N*-dimethylacetamide, DME = 1,2-dimethoxyethane, MLM = mechanoluminescent materials, DABCO = 1,4-diazabicyclo[2.2.2]­octane, TEA = triethylamine, RAM = resonant acoustic mixer, 4CzIPN = 1,2,3,5-tetrakis­(carbazol-9-yl)-4,6-dicyanobenzene.

## Future Research Frontiers

The field of mechanophotocatalysis has developed rapidly in recent years, and it is becoming evident that this methodology can be employed to drive a wide range of photocatalysis reactions. Furthermore, we have seen evidence that there can be additional benefits gained from using this approach aside from minimizing the amount of solvent used.^55,56^ Given the recent acceleration of new publications in this area, we anticipate that this methodology will receive increasing attention over the next few years, with research likely directed toward a number of key areas (Figure 5a). We expect this approach will be expanded toward a wider range of photocatalysis reactions, cementing its generality and increasing the confidence of the synthetic community that this is a valuable tool for both academic laboratories and for industry. We also anticipate the development of new enabling reactor technologies^86^ that facilitate high-throughput screening of reactions and reaction scale-up. In this context, RAM reactors have shown particular promise, as these platforms can be used for both applications in the same reactor (for example, Rueping and co-workers could conduct reactions at a range of scales from 0.4 to 300 mmol).^62^ However, RAM reactors are much more expensive than ball mills; thus, both technologies are likely to play important roles in the continuing development of mechanophotocatalysis. Noting that reactivity is ostensibly driven by light, and the mechanical agitation is primarily responsible for mixing the reagents, we also anticipate the development of lower energy mechanochemical grinding approaches. Temperature control will be important for new reactor designs. Higher temperatures are likely to contribute to the melting of reaction components to facilitate more intimate mixing, and may be beneficial in reaction systems containing transition metal cocatalysts;^53,87^ however, lower temperatures may be necessary in reactions requiring precise selectivity control.^88^ Thus, reactors that effectively monitor and control temperature are needed.

**Figure 5:**
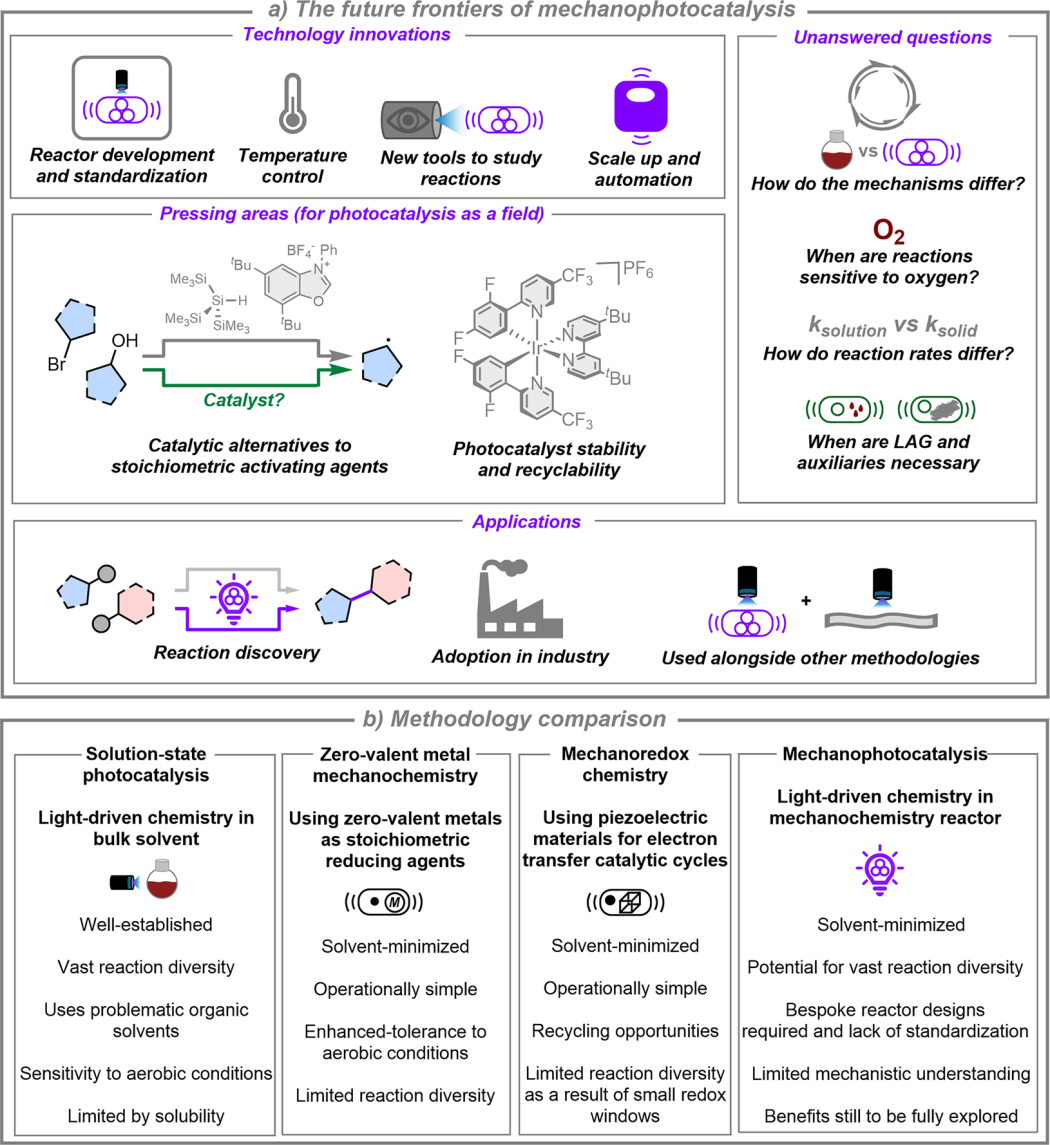
a) Anticipated frontiers for future mechanophotocatalysis research. b) Comparing the benefits and pitfalls of different synthetic methodologies: solution-state photocatalysis, mechanochemistry with zerovalent metals, mechanoredox chemistry, and mechanophotocatalysis.

Alongside the development of new reactor technologies, a complementary approach pioneered by Wu, Wang and co-workers is the use of mechanoluminescent materials as internal light sources.^83^ If such a methodology could be extended to reactions of industrial relevance, then its potential impact would increase significantly. Such an approach would obviate the requirement for using transparent reaction vessels, and potentially enable the more facile scale-up of light-initiated reactions using existing mechanochemistry tools, such as twin screw extruders.^89^ To achieve this potential, efforts will likely need to be directed toward (1) exploitation of high-energy (blue-light emitting) mechanoluminescent materials that would enable a wider spectrum of photochemical reactions to be realized; and (2) confirmation of whether emission from such mechanoluminescent materials is present in different grinding environments such as those of twin screw extruders and resonant acoustic mixers.

Another potential advantage of mechanophotocatalysis is accelerating reaction optimization workflows by removing the consideration of solvent choice. However, throughout our work using ball mill reactors, we have found that the rheology of mechanophotocatalysis reaction mixtures is intimately linked with reaction efficiency, and optimizing this can also be time-consuming.^55–57^ Evidently, the roles of grinding auxiliaries and LAG agents, and the situations for when these additives are necessary, should also be explored further. Mechanophotocatalysis also offers the potential to expand the accessible chemical space by using poorly soluble (or insoluble) reaction components, as demonstrated in our proof-of-concept studies with sodium ascorbate.^57^ The expansion of this concept to other substrates and catalysts that are poorly soluble in organic solvents could be particularly advantageous. Furthermore, thus far we have assumed that light drives the observed reactivity, and mechanochemistry is only responsible for mixing reactants and generating new reactive surfaces. However, it is not inconceivable that reactions could be developed that utilize both mechanochemical and photochemical energy within the same system; for example, using nickel-coated balls for a direct metallaphotoredox catalysis protocol.^90^

Greater insight into mechanophotocatalysis reaction mechanisms, and how these may diverge from reactions conducted in solution is also of key importance. Crucially, while some reports demonstrate that mechanophotocatalysis can provide an enhanced tolerance to aerobic conditions over solution-state conditions,^55,56^ other systems have required an inert atmosphere,^62,83^ and the origins for these diverging results must be identified. Mechanochemistry is readily amenable to reactions with gaseous components,^91,92^ and aerobic oxidations have been reported under mechanophotocatalysis conditions;^59,72^ however, these aerobic mechanophotocatalysis reactions did require extended reaction times, suggesting slow kinetics. Clearly, more insight is needed to probe the kinetics of photochemical reactions at the gas/solid interface. More broadly, studies quantifying reaction rate comparisons between solution and mechanophotocatalysis versions of the same reaction would be of value. New tools for studying the mechanisms of solvent-minimized photochemical reactions will also need to be developed. In particular, *in situ* monitoring using Raman and emission spectroscopies will likely be key for studying mechanophotocatalysis systems.^60,80,93^

As with all new methodologies, the potential limitations of mechanophotocatalysis must be explored to enable chemists to decide which photocatalysis methodology would be best suited to a particular objective. For example, solution-state continuous flow photoreactors are likely better suited for mediating reactions with hazardous chemicals or reactions involving gaseous reagents than a mechanophotocatalysis approach.^86^ On the other hand, mechanophotocatalysis should be used to mediate heterogeneous reactions that are difficult to adapt to flow reactors, thus enabling complementary use cases between these two technologies.

We are excited to witness the wider uptake of mechanophotocatalysis by both academia and industry. This field is developing alongside the broader implementation of other mechanochemistry processes in industry,^94^ and has significant potential to increase the sustainability of industrial processes.^95^ For mechanophotocatalysis to be successfully deployed in industry, several specific challenges must be overcome. This includes the standardization of reactor technologies, which would enable reliable comparisons to be made between laboratories. Safety assessments will need to be made during the validation of this approach in an industrial setting,^96^ and the challenge of how to automate this chemistry will be of particular relevance for HTE applications. Mechanophotocatalysis will need to be shown to be a competitive synthetic methodology across a wider range of reactions for it to be considered as the first option for the industrial chemist.

As photocatalysis (both in the solution-state and under solvent-minimized conditions) becomes increasingly used for larger-scale industrial processes,^1^ we foresee two bottleneck issues that should be addressed. First, many of the most valuable metallaphotoredox catalysis reactions rely on stoichiometric reagents, such as *Deoxazole* and tris­(trimethylsilyl)­silane, to activate widely available coupling partners such as alkyl alcohols and halides,^5–7,51,97–99^ leading to the stoichiometric formation of byproducts that create significant waste and hinders product purification. Currently, catalytic alternative methods for activating these functional groups have much more limited substrate compatibility than state-of-the-art stoichiometric additive systems.^100,101^ Thus, the development of new catalytic systems for the activation of these functional groups in the context of metallaphotoredox catalysis reactions would be highly desired for the implementation of these reactions in industry at scale, be it in the solution-state or using mechanophotocatalysis methodologies. Second, the issue of photocatalyst instability/degradation is a universal problem that reduces catalyst turnover numbers, limits their recyclability, complicates purification, and makes the prediction of an optimal photocatalyst for a transformation problematic.^102^ The development of stable photocatalysts is key to addressing these issues.

Mechanophotocatalysis will need to be shown to be a competitive synthetic methodology across a wider range of reactions for it to be considered as the first option for the industrial chemist.

Conceptually, mechanophotocatalysis is similar to zerovalent metal mechanochemistry^38^ and mechanoredox chemistry^103^ as all three synthesis methodologies involve single electron transfer chemistry, Figure 5b. These approaches involve milling of either zerovalent metals (which can facilitate a range of net-reductive transformations) or piezoelectric materials (which become polarized upon mechanical agitation to facilitate reactivity similar to the oxidative quenching cycle of a photocatalyst). These alternative solvent-minimized electron transfer reaction methodologies are, from a practical perspective, simple to implement, as conventional (nontransparent) mechanochemistry tools can be used. However, the reaction diversity demonstrated by these approaches is significantly more limited than light-driven processes due in part to the tunability of the photocatalysts. Ultimately, each of these tools has value for different targeted synthetic transformations and should continue to be developed in tandem.

Despite still being a relatively new field, it is impressive how rapidly mechanophotocatalysis has developed in such a short time, and it is undeniable that there is a bright horizon for future research ahead.

## Supplementary Material



## Notes

Transparent Peer Review report available (PDF)
