# Supplementary material for: Shaking Up Photochemistry: The Future Frontiers of Mechanophotocatalysis
Source: ACS Cent Sci. 2026 Jan 1;12(1):17–27. doi: 10.1021/acscentsci.5c01770 (PMC12871882; doi:10.1021/acscentsci.5c01770)
Supplement: Supplementary file 1 [file oc5c01770_si_001.pdf]

oc-2025-01770g.R1

Name: Peer Review Information for "Shaking Up Photochemistry: The Future Frontiers of Mechanophotocatalysis"

First Round of Reviewer Comments

Reviewer: 1

Comments to the Author

The authors present an outlook on the emerging field of mechanophotocatalysis, a hybrid approach that combines photocatalysis with mechanochemistry to perform solvent-minimized, light-driven reactions. Millward and Zysman-Colman highlight the environmental and operational limitations of traditional solution-state photocatalysis—particularly the heavy reliance on toxic and flammable solvents—and propose mechanophotocatalysis as a sustainable alternative. They review recent developments in the field, showcase examples where this methodology has improved reaction efficiency and oxygen tolerance, and outline future research directions including reactor design, mechanistic studies, and industrial scalability. The article underscores mechanophotocatalysis as a promising complementary tool to conventional photochemistry for synthetic organic chemistry under greener conditions.

While the manuscript is highly informative and well-written, I believe there are several areas where further clarification or elaboration would enhance its impact and accessibility. These are detailed below.

1. Role of the Beer–Lambert Law in Photochemical Dilution

The authors mention the Beer–Lambert Law in the context of scale-up limitations but should go further to explain how this law inherently necessitates the use of diluted solutions in classical photochemistry. These dilute conditions directly lead to problems such as slow kinetics, large solvent volumes, and inefficient light utilization. Making this connection explicit would help frame mechanophotocatalysis as a direct solution to a foundational limitation in the field.

2. Underrepresentation of Mechanoluminescent Materials in Future Outlook

Mechanoluminescent materials are briefly acknowledged, yet their broader potential as photon sources within opaque or sealed systems is not fully explored. This technology bypasses the need for transparent reaction vessels and could offer a low-barrier method for implementing photochemistry in solid-state settings. Given the theme of the Outlook, a more thorough inclusion in the "Future Frontiers" section is warranted.

3. Limited Treatment of Industrial Relevance

Although the term “industry” appears multiple times, the manuscript lacks a detailed discussion of mechanophotocatalysis in an industrial context. A comparative assessment with more established methods—particularly photo-flow chemistry—regarding costs, scalability, reliability, and regulatory considerations would be highly valuable for both academic and industrial readers.

4. Lack of Discussion on Irradiation Methods

While reactor and vessel developments are nicely summarized, the evolution of irradiation methodologies is largely overlooked. A brief commentary on the progression from early, rudimentary light setups (e.g., wrapping LEDs around jars) to modern integrated photoreactors would complete the picture. This would also better inform readers about the practical challenges and solutions encountered in setting up mechanophotocatalysis reactions.

5. Superficial Comparison to Other Mechanochemical Approaches

The comparison of mechanophotocatalysis with other solvent-free electron transfer methods such as piezoelectric mechanoredox and zero-valent metal mechanochemistry is intriguing but underdeveloped. Currently, it is covered in just a few sentences before the conclusion. Given the conceptual relevance and potential for cross-fertilization between these approaches, the authors should either expand this comparison meaningfully or remove it altogether to maintain the manuscript’s depth and focus.

6. Figure 2 Layout and Design

While informative, Figure 2 suffers from visual imbalance and clutter due to non-uniform box sizes and dense labeling. The figure would benefit from consistent formatting and possibly a reorganization by reaction type or reactor platform to improve legibility.

7. Figure 3: Typos and Conceptual Cohesion

Typos in Figure 3 should be corrected:

o “Photocatalyt stability” → Photocatalyst stability

o “oppertunities” → opportunities

o “Enhanced-tolerence” → Enhanced tolerance

Beyond the typographical issues, the “Key Questions” section of the figure introduces important ideas that are not thoroughly discussed in the main text. This mismatch may confuse readers. Either these questions should be more fully addressed, or the figure should be aligned more closely with the manuscript’s content.

Reviewer: 2

Comments to the Author

In this Perspective, Zysman-Colman and Francis Millward present an overview of recent examples of mechanophotocatalytic transformations. The existence of a recent review on the same topic (ref. 52) should not necessarily be regarded as a drawback, provided that the authors clearly contextualize their contribution and offer a balanced comparison. Even though the narrative is different, it is currently unclear which of the examples discussed here overlap with those in ref. 52 and which represent new additions. As the authors themselves note, this is a rapidly evolving field, with numerous publications appearing even within the past year. Even highlighting only these reports, perhaps by modifying the title to something like *“Recent advancements in mechanophotocatalysis”*, would not affect the scope and relevance of the manuscript.

Other than that, I found it an engaging discussion on an area that is attracting increasing attention due to its unique ability to combine the principles of mechanochemistry and photochemistry. This work deserves publication after the following comments have been addressed:

- When mentioning the activation of zero-valent metals, one could add recent developments in the field including DOI: 10.1002/cssc.202500211 and 10.1002/anie.202405342.
- As it often happens with emerging fields, new terms are advanced. An explanation on why “mechanophotocatalysis” should be adopted over previously proposed ones should be provided.
- In Figure 1C, typo: “solubalises” to “solubilizes”.

- On page 6, line 38, there are some repetitions in the text. To improve fluency and readability, the sentence could be streamlined by writing, for example: “Two years later, the same group reported…” rather than repeating the author’s name.
- On page 8, line 19, the explanation regarding resonant acoustic mixing (RAM) could be expanded for readers that are less familiar with this technology. While specialists in the field may immediately recognize the differences between RAM vessels and classical jars, those without direct experience may find the explanation somewhat vague.

In summary, this perspective represents a valuable contribution to the burgeoning field of mechanophotocatalysis. The suggested clarifications will further strengthen the manuscript, making it more accurate, accessible, and complete.

Reviewer: 3

Comments to the Author

This Outlook presents an engaging and comprehensive perspective on a highly dynamic and emerging area at the interface of photochemistry and mechanochemistry. The authors succeed in clearly articulating the motivation for merging these two methodologies, emphasizing how mechanophotocatalysis can address long-standing limitations of solution-state photocatalysis, such as solvent waste, flammability, oxygen sensitivity, and limited solubility of substrates, while opening new synthetic and technological opportunities. The paper is well structured and offers a logically progressive discussion that spans fundamental concepts, recent developments, and forward-looking insights into reactor design, mechanistic understanding, and future applications.

The overview of past and recent advances is impressively complete, capturing the rapid evolution of this field and highlighting key methodological milestones ranging from early thin-film photochemical studies to more sophisticated ball mill and resonant acoustic mixing approaches. The emphasis on the interplay between photochemical reactivity and mechanical mixing is particularly timely and relevant. Furthermore, the discussion of potential advantages, such as enhanced aerobic tolerance and the ability to employ poorly soluble substrates, helps position mechanophotocatalysis as a genuine alternative and complement to classical photochemical methods.

While the manuscript is already strong, I have only minor recommendations.

1) It could benefit from a short paragraph discussing the scope and limitations of mechanophotocatalysis, particularly regarding when solvent participation remains essential, for example, in reactions where solvents influence selectivity, moderate exothermicity, or tune reaction kinetics. Such context would sharpen the reader's understanding of where the technique is most promising and where further technological or conceptual development is still required.

2) Additionally, referencing the recent review J. Am. Chem. Soc. 2025, 147, 32, 28523–28545 would be valuable, as it provides a topical complement to this Outlook and situates the discussion within the latest literature.

Overall, this is a thoughtful, well-written, and forward-looking contribution that is likely to attract attention and stimulate further research in this exciting emerging field. It provides a clear and accessible roadmap for both chemists entering the area and those seeking to understand how mechanophotocatalysis may reshape sustainable photochemical synthesis in the years ahead.

Reviewer: 4

Comments to the Author

The manuscript proposed as an Outlook article by Millward and Zysman-Colman is focused on recent developments and prospects of mechanophotocatalysis as an emerging methodology within the realm of solid state mechanochemistry. The authors have listed important publications in the field in a chronological order, showing how photocatalysis in the solid state has evolved from the first simple rod mill-type setup to vibrational ball milling and resonant acoustic mixing, as well as the increase in complexity of organic transformations carried out under these conditions. As a relatively new addition to the mechanochemical toolbox, mechanophotocatalysis is shown as a promising alternative to solution-based photocatalytic reactions, and disscussed in terms of potential niches for future investigations. As such, it is expected that research in mechanophotocatalysis will gain momentum as our understanding of symbiosis of milling and photochemistry

broadens in the years to come. The manuscript itself is well written and is suitable for publication in ACS Central Science after minor revision:

1. Page 2, line 25: "... due to its ability to mediate solvent-minimized versions of known solution-state reactions".

Not only this, mechanochemistry allows access to organic molecules not stable in solution environment (reactive intermediates) as well as the discovery of solid-state reactivity different from the one observed in solvents. Refs might include Angew. Chem. Int. Ed. 2015, 54, 8440. and Angew. Chem. Int. Ed. 2014, 53, 9321.

2. Page 2, lines 26, 28 and Figure 1: I'm not sure that the term "transmutation" is the most appropriate one to describe the use of mechanochemistry in place of conventional solution-based approach.

3. Page 4, line 5: "...while others have used the terms solid-state photochemistry,49,50 or photomechanochemistry.51-53"

For the sake of accuracy, the term "solid-state photochemistry" is used in ref 49 and "mechanochemically-assisted solid-state photocatalysis" in ref 50. Please refer to this as well.

4. Page 4, line 44: Besides chemical resistivity, the opacity of PMMA jars represents another obstacle for an efficient photochemical reaction to proceed.

5. Page 5, Figure 2. In my opinion, the figure would probably work better if it was split into several figures, each placed in the respective paragraphs. In this way, it would be much easier for the reader to follow the text and see the transformations discussed. Please take into consideration.

6. Page 6, line 26: I suggest the sentence "In 2017,..." starts with a new paragraph.

7. Page 6, lines 29 and 32: The reactions described in refs 50 and 62 were photocatalytic and used Eosin Y as a photocatalyst, this should be added. Also, ref 50 reported on the use

of LED strips for merging photocatalysis and ball milling, as well as on the design of a LED photoreactor compatible with simultaneous ball millng. In ref 62, LED strip was employed. These two approaches have later been adopted (with minor modifications) by other research groups and thus represent an important contribution to the field of mechanophotocatalysis. Therefore I suggest that a scheme is inserted here showing these two designs, as they are easy to implement and readers may find it useful for their research.

8. Page 7, line 25: In relation to my previous comment, it would be also nice to see the reaction vessel design from the author's group. This could be included in the same scheme with LED reactor/strip or as a separate scheme.

9. Page 8, line 34: What is 4CzIPN? Please provide a full chemical name.

10. Page 9, line 16: RAM reaction scale up - what is the typical scale of these reactions in RAM?

11. Page 10, line 23: "a" should be deleted.

12. Page 10, line 26: A reference for large-scale industrial photocatalytic processes should be placed here.

13. Page 11, Figure 3. I suggest to add "scale up" under Technology innovations, as an important aspect of full implementation of mechanophotocatalysis in the future.

Author's Response to Peer Review Comments:

ELI ZYSMAN-COLMAN, Ph.D. **School of Chemistry, University of St Andrews,**
Purdie Building, North Haugh
KY16 9ST
eli.zysman-colman@st-andrews.ac.uk
Tel.: +44-1334 463826; Fax.: +44-1334 463808 http://www.zysman-colman.com

25/11/2025

Dear Editor,

Please find enclosed our revised invited manuscript (oc-2025-01770g) entitled "**Shaking Up Photochemistry: The Future Frontiers of Mechanophotocatalysis**" co-authored by Francis Millward and me. We believe that we have now adequately addressed all of the reviewers' comments as well as those from your office. We provide a highlighted version of the manuscript and a point-by-point response below. We hope that the current version is acceptable for publication.

Formatting Needs:

Pull Quotes (Outlook): We encourage you to select 3 - 4 quotes from your Outlook that you would like highlighted in your paper. The quotes should be one sentence-long, unique to the Outlook and not from previously cited work. Please list your quotes at the end of the manuscript file.

Our response: The following quotes have been listed at the end of the article:

'Despite their apparently simple role as reaction media, solvent represents the greatest contributor to the total mass in a typical reaction system, and becomes the largest source of waste throughout a multistep route.'

'Considering the solvent-associated issues for conducting photocatalysis reactions, and the apparent benefits of adapting reactions to a mechanochemical environment, it is apparent that the successful merger of these two complementary methodologies could lead to a paradigm shift in how light-driven reactions are conducted.'

'Therefore, the most effective approach for developing a generalizable protocol for solvent-minimized photocatalysis reactions likely involves simultaneous mechanical agitation and light irradiation.'

'Mechanophotocatalysis will need to be shown to be a competitive synthetic methodology across a wider range of reactions for it to be considered as the 1st option for the industrial chemist.'

Synopsis: ACS Central Science requires a brief synopsis. The synopsis should be no more than 200 characters (including spaces) and should reasonably correlate with the Table of Contents (TOC) graphic. The synopsis is intended to explain the importance of the article to a broader readership across the sciences. Please place your synopsis in the manuscript file after the TOC graphic and label as "Synopsis."

Our response: We have added the following synopsis:

> 'Light-driven reactions, facilitated by mechanical mixing, have the potential to revolutionize photochemistry.
>
> This Outlook explores the development and potential applications of mechanophotocatalysis.'

Reviewer(s)' Comments to Author:

Reviewer: 1

Recommendation: Publish in ACS Central Science after minor revisions noted.

Comments:
The authors present an outlook on the emerging field of mechanophotocatalysis, a hybrid approach that combines photocatalysis with mechanochemistry to perform solvent-minimized, light-driven reactions. Millward and ZysmanColman highlight the environmental and operational limitations of traditional solution-state photocatalysis—particularly the heavy reliance on toxic and flammable solvents—and propose mechanophotocatalysis as a sustainable alternative. They review recent developments in the field, showcase examples where this methodology has improved reaction efficiency and oxygen tolerance, and outline future research directions including reactor design, mechanistic studies, and industrial scalability. The article underscores mechanophotocatalysis as a promising complementary tool to conventional photochemistry for synthetic organic chemistry under greener conditions.
While the manuscript is highly informative and well-written, I believe there are several areas where further clarification or elaboration would enhance its impact and accessibility. These are detailed below.

Our response: We thank the reviewer for their positive comments and support of the manuscript, as well as for their insightful suggestions. We have addressed each of these suggestions below.

1. Role of the Beer–Lambert Law in Photochemical Dilution

The authors mention the Beer–Lambert Law in the context of scale-up limitations but should go further to explain how this law inherently necessitates the use of diluted solutions in classical photochemistry. These dilute conditions directly lead to problems such as slow kinetics, large solvent volumes, and inefficient light utilization. Making this connection explicit would help frame mechanophotocatalysis as a direct solution to a foundational limitation in the field.

Our response: The reviewer makes an important note here that we should have elaborated on. We have added additional text in the '*Photocatalysis: the solvent problem'* section to address this on page 2:

> 'Furthermore, solution-state photochemical reactions are difficult to scale in batch, mainly due to inefficient light penetration in larger vessels as a result of the Beer-Lambert Law, which describes the exponential attenuation of light intensity through a reaction mixture as a function of increasing path length and concentration of the light-absorbing materials. As a result, photochemical reactions are often conducted as dilute solutions, which increases solvent waste and leads to nonuniform irradiation and slower reaction kinetics within batch reactors.[16]'

2. Underrepresentation of Mechanoluminescent Materials in Future Outlook

Mechanoluminescent materials are briefly acknowledged, yet their broader potential as photon sources within opaque or sealed systems is not fully explored. This technology bypasses the need for transparent reaction vessels and could offer a low-barrier method for implementing photochemistry in solid-state settings. Given the theme of the Outlook, a more thorough inclusion in the "Future Frontiers" section is warranted.

Our response: We thank the reviewer for this suggestion; this is indeed an exciting area for future development, particularly in the endeavour to use these materials for blue light-driven reactions, and for their potential use in metallaphotoredox catalysis reactions, which would have significant value. To address this suggestion, we have included the following text in the future frontiers section on page 12:

‘Alongside the development of new reactor technologies, a complementary approach pioneered by Wu, Wang and co-workers is the use of mechanoluminescent materials as internal light sources. If such a methodology could be extended to reactions of industrial relevance, then its potential impact would increase significantly.[87] Such an approach would obviate the requirement for using transparent reaction vessels, and potentially enable the more facile scale-up of light-initiated reactions using existing mechanochemistry tools, such as twin screw extruders.[88] To achieve this potential, efforts will likely need to be directed towards (1) exploitation of high-energy (blue-light emitting) mechanoluminescent materials that would enable a wider spectrum of photochemical reactions to be realized; and (2) confirmation of whether emission from such mechanoluminescent materials is present in different grinding environments such as those of twin screw extruders and resonant acoustic mixers.’

3. Limited Treatment of Industrial Relevance

Although the term “industry” appears multiple times, the manuscript lacks a detailed discussion of mechanophotocatalysis in an industrial context. A comparative assessment with more established methods— particularly photo-flow chemistry—regarding costs, scalability, reliability, and regulatory considerations would be highly valuable for both academic and industrial readers.

Our response: We thank the reviewer for this suggestion. We have now dedicated a short paragraph to explicitly target the potential challenges that face mechanophotocatalysis when being adapted to an industrial setting on page 14:

‘We are excited to witness the wider uptake of mechanophotocatalysis by both academia and industry. This field is developing alongside the broader implementation of other mechanochemistry processes in industry,[92] and has significant potential to increase the sustainability of industrial processes.[93] For mechanophotocatalysis to be successfully deployed in industry, several specific challenges must be overcome. This includes the standardization of reactor technologies, which would enable reliable comparisons to be made between laboratories. Safety assessments will need to be made during the validation of this approach in an industrial setting,[94] and the challenge of how to automate this chemistry will be of particular relevance for HTE applications. Mechanophotocatalysis will need to be shown as a competitive synthetic methodology across a wider range of reactions for it to be viewed as a 1st option for the industrial chemist.’

4. Lack of Discussion on Irradiation Methods

While reactor and vessel developments are nicely summarized, the evolution of irradiation methodologies is largely overlooked. A brief commentary on the progression from early, rudimentary light setups (e.g., wrapping LEDs around jars) to modern integrated photoreactors would complete the picture. This would also better inform readers about the practical challenges and solutions encountered in setting up mechanophotocatalysis reactions.

Our response: This is a good suggestion. We have modified much of the literature overview section to describe the types of light sources that have been used in each case, with these changes highlighted throughout the 'Mechanophotocatalysis: Development and Trajectory' section.

5. Superficial Comparison to Other Mechanochemical Approaches

The comparison of mechanophotocatalysis with other solvent-free electron transfer methods such as piezoelectric mechanoredox and zero-valent metal mechanochemistry is intriguing but underdeveloped. Currently, it is covered in just a few sentences before the conclusion. Given the conceptual relevance and potential for cross-fertilization between these approaches, the authors should either expand this comparison meaningfully or remove it altogether to maintain the manuscript's depth and focus.

Our response: We understand the reviewer's point. In our original version, we had placed much of the comparison of these methodologies within the Figure, while keeping the main text portion brief. We believe that the inclusion of this comparison has value to the readers and gives more context to alternative methodologies that are all ultimately complementary to one another, and therefore, we believe that keeping this discussion in the article is important. However, as this is a perspective on mechanophotocatalysis, we wanted to keep this section concise. As a compromise, we have expanded the section slightly to explain how each methodology works, and list some of the advantages and disadvantages that are also highlighted in the Figure:

'Conceptually, mechanophotocatalysis is similar to zero-valent metal mechanochemistry[38] and mechanoredox chemistry[103] as all three synthesis methodologies involve single electron transfer chemistry, Figure 5b. These approaches involve milling of either zero-valent metals (which can facilitate a range of net-reductive transformations) or piezoelectric materials (which become polarized upon mechanical agitation to facilitate reactivity similar to the oxidative quenching cycle of a photocatalyst). These alternative solvent-minimized electron transfer reaction methodologies are, from a practical perspective, simple to implement, as conventional (non-transparent)

mechanochemistry tools can be used. However, the reaction diversity demonstrated by these approaches is significantly more limited than light-driven processes due in part to the tunability of the photocatalysts. Ultimately, each of these tools has value for different targeted synthetic transformations and should continue to be developed in tandem.'

6. Figure 2 Layout and Design

While informative, Figure 2 suffers from visual imbalance and clutter due to non-uniform box sizes and dense labeling. The figure would benefit from consistent formatting and possibly a reorganization by reaction type or reactor platform to improve legibility.

Our response: We agree that this Figure is too crowded; we had hoped to concisely show all the current examples in one place as a reference for readers, but we recognise that in its current form, the figure likely will be overwhelming for the reader. To address this issue, we have converted this figure into three separate single column figures grouped broadly by reaction class and approach:

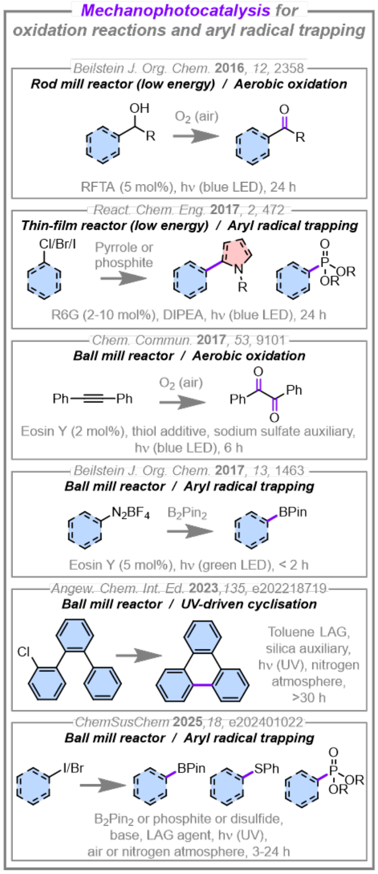

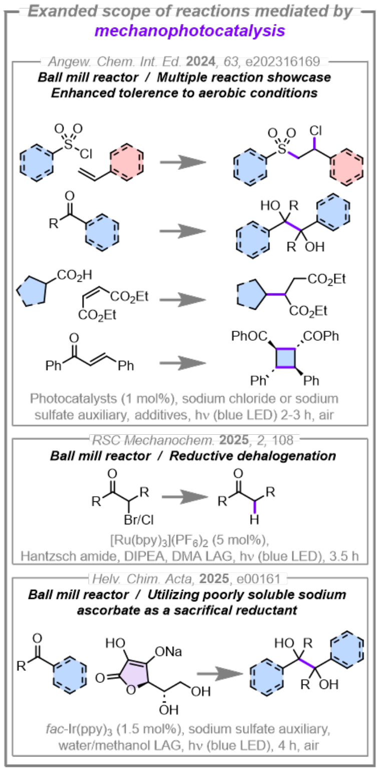

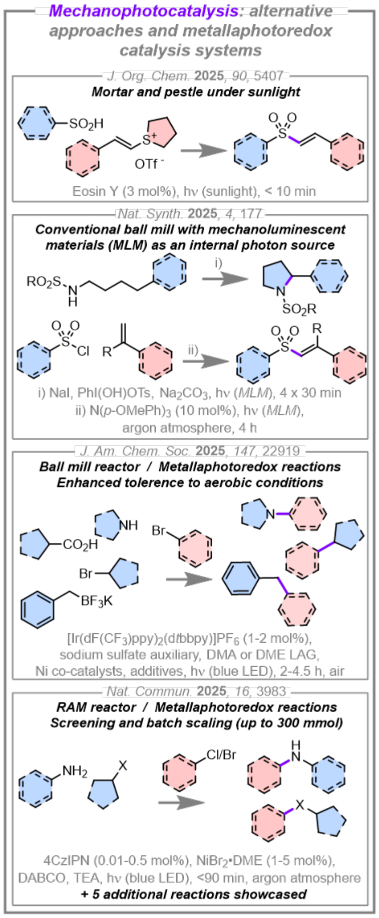

7. Figure 3: Typos and Conceptual Cohesion Typos in Figure 3 should be corrected: o "Photocatalyt stability" → Photocatalyst stability o "oppertunities" → opportunities
o "Enhanced-tolerence" → Enhanced tolerance
Beyond the typographical issues, the "Key Questions" section of the figure introduces important ideas that are not thoroughly discussed in the main text. This mismatch may confuse readers. Either these questions should be more fully addressed, or the figure should be aligned more closely with the manuscript's content.

Our response: We thank the reviewer for catching these issues. We have corrected the typographical errors. We have also modified the entire figure to more closely align with the challenges outlined in the main text:

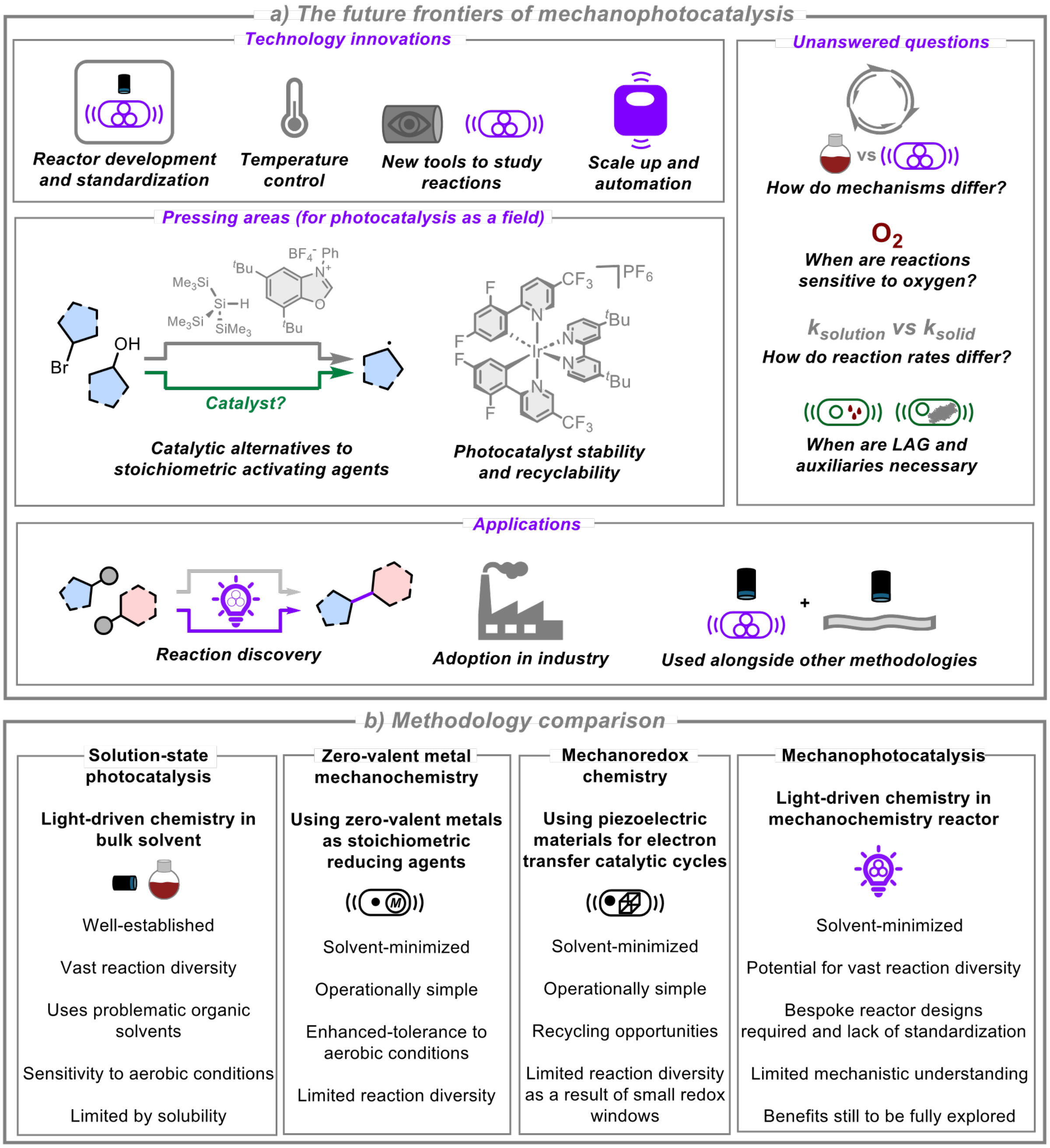

Reviewer: 2

Recommendation: Major revisions required.

Comments:
In this Perspective, Zysman-Colman and Francis Millward present an overview of recent examples of mechanophotocatalytic transformations. The existence of a recent review on the same topic (ref. 52) should not necessarily be regarded as a drawback, provided that the authors clearly contextualize their contribution and offer a balanced comparison. Even though the narrative is different, it is currently unclear which of the examples discussed here overlap with those in ref. 52 and which represent new additions. As the authors themselves note, this is a rapidly evolving field, with numerous publications appearing even within the past year. Even highlighting only these reports, perhaps by modifying the title to something like "Recent advancements in mechanophotocatalysis", would not affect the scope and relevance of the manuscript.

Our response: We appreciate the reviewer's point. We do mention explicitly that the said review only includes examples until the end of 2024, and since then, there have been several valuable advances in the field, including the use of RAM and the demonstration of metallaphotoredox reactions. In total, 10 new reports were published in the last year alone, and this has led to a practical doubling of the number of examples relevant for synthetically useful reactions. However, we do not believe that it is appropriate to only discuss the examples that have been released since the end of 2024, as these advances must be contextualised in the presence of the previous work, as the field is still very much in its infancy. Our narrative on the motivation for using mechanophotocatalysis, its development in terms of the types of reactions that have been disclosed, and future directions, is also distinct (and we think complementary to) the previous review, which has much more of a focus on the specific mechanical technologies that have been previously used.

To make this distinction more explicit, we have modified the text at the end of the 'Mechanophotocatalysis: The Design Principle' section on page 4:

'A recent review documents literature examples up to the end of 2024;[59] however, since then, there have been several important advances in the field, including more industrially relevant reactions being adapted to solventminimized conditions, and RAM being used to mediate these processes. To facilitate the continuing acceleration of research in this exciting new field, we summarize key advances for applications in organic synthesis (Figures **2**, **3**, and **4**), providing context for a discussion of how we anticipate this rapidly evolving methodology will develop in the future.'

Other than that, I found it an engaging discussion on an area that is attracting increasing attention due to its unique ability to combine the principles of mechanochemistry and

photochemistry. This work deserves publication after the following comments have been addressed:

Our response: We thank the reviewer for this support and we appreciate their recommendations for additions to our manuscript.

- When mentioning the activation of zero-valent metals, one could add recent developments in the field including DOI:
10.1002/cssc.202500211 and 10.1002/anie.202405342.

Our response: We thank the reviewer for bringing these articles to our attention. We were aware of these papers and thoroughly enjoyed reading these valuable contributions to the field. However, this perspective remains focused on mechanophotocatalysis, and we have included only a small mention of mechanoredox and zero valent mental mechanochemistry as a way of making uninitiated readers aware of the alternative methodologies present for these fields. As such, we have deliberately only referenced one review article for each application (Chem. Eur. J. 2021, 27, 9721-9726 and Nat. Synth. 2022, 1, 763-775 for each field, respectively). We have thus chosen not to cite additional specific examples of work in the field.

- As it often happens with emerging fields, new terms are advanced. An explanation on why "mechanophotocatalysis" should be adopted over previously proposed ones should be provided.

Our response: Since the reactivity of these transformations is driven by light (and often photocatalysts), we believe that 'mechanically mediated photocatalysis' and 'mechanophotocatalysis' are more appropriate terms in general than 'photomechanochemistry', which leans slightly towards inferring the reactions are principally mechanochemical in nature. We have added this to the text on page 4:

'A small number of reports exist that document the use of different mechanochemistry tools for mediating photochemical reactions in the absence of bulk solvent. We refer to this field as *mechanophotocatalysis,* as this term encapsulates a chemistry that is mediated by light, while the mechanochemistry is responsible for mixing,[53-55] while others have used the terms *solid-state photochemistry*,[56] *mechanochemically-assisted solid-state photocatalysis*,[57] or *photomechanochemisty*.[58-60]'

- In Figure 1C, typo: “solubalises” to “solubilizes”.

Our response: We thank the reviewer for noticing this issue, which has now been corrected.

- On page 6, line 38, there are some repetitions in the text. To improve fluency and readability, the sentence could be streamlined by writing, for example: “Two years later, the same group reported…” rather than repeating the author’s name.

Our response: We thank the reviewer for this suggestion, which we have implemented.

- On page 8, line 19, the explanation regarding resonant acoustic mixing (RAM) could be expanded for readers that are less familiar with this technology. While specialists in the field may immediately recognize the differences between RAM vessels and classical jars, those without direct experience may find the explanation somewhat vague.

Our response: This is an excellent point. We have updated the text to give a more complete description of RAM as a methodology:

‘Recently, RAM has been used to drive solvent-minimized photochemical reactions. In contrast to ball milling, which uses milling balls to grind and mix reactions, RAM induces mechanical mixing by the vertical oscillation of the reaction vessel at a set frequency (typically 60 Hz), with the energy of the system being modulated by varying the amplitude of the oscillation.[82, 83] The induced acoustic energy enables the rapid mixing of the reaction contents in the complete absence of milling media, meaning glass reaction vessels can be used, and the reaction setup is simplified. Consequently, this ability to use standard laboratory glassware in a mechanophotocatalysis setting obviates the need for bespoke reaction vessels.’

In summary, this perspective represents a valuable contribution to the burgeoning field of mechanophotocatalysis. The suggested clarifications will further strengthen the manuscript, making it more accurate, accessible, and complete.

Our response: We thank the reviewer for their support of this work and their suggestions, and we hope that the implementations we have made are satisfactory.

Reviewer: 3

Recommendation: Publish in ACS Central Science after minor revisions noted.

Comments:
This Outlook presents an engaging and comprehensive perspective on a highly dynamic and emerging area at the interface of photochemistry and mechanochemistry. The authors succeed in clearly articulating the motivation for merging these two methodologies, emphasizing how mechanophotocatalysis can address long-standing limitations of solution-state photocatalysis, such as solvent waste, flammability, oxygen sensitivity, and limited solubility of substrates, while opening new synthetic and technological opportunities. The paper is well structured and offers a logically progressive discussion that spans fundamental concepts, recent developments, and forward-looking insights into reactor design, mechanistic understanding, and future applications.

The overview of past and recent advances is impressively complete, capturing the rapid evolution of this field and highlighting key methodological milestones ranging from early thin-film photochemical studies to more sophisticated ball mill and resonant acoustic mixing approaches. The emphasis on the interplay between photochemical reactivity and mechanical mixing is particularly timely and relevant. Furthermore, the discussion of potential advantages, such as enhanced aerobic tolerance and the ability to employ poorly soluble substrates, helps position mechanophotocatalysis as a genuine alternative and complement to classical photochemical methods. While the manuscript is already strong, I have only minor recommendations.

Our response: We thank the reviewer for their enthusiasm and positive support of the Outlook, and we will endeavour to address their recommendations as completely as possible below.

1) It could benefit from a short paragraph discussing the scope and limitations of mechanophotocatalysis, particularly regarding when solvent participation remains essential, for example, in reactions where solvents influence selectivity, moderate exothermicity, or tune reaction kinetics. Such context would sharpen the reader's understanding of where the technique is most promising and where further technological or conceptual development is still required.

Our response: The reviewer makes an excellent suggestion. Accordingly, we have dedicated a short section discussing the need to explore the potential limitations of

mechanophotocatalysis and impressing upon the reader that alternative methodologies (such as flow chemistry) may be more appropriate techniques for different situations:

‘As with all new methodologies, the potential limitations of mechanophotocatalysis must be explored provide the chemist with information needed to decide which photocatalysis methodology would be best suited to a particular objective. For example, solution-state continuous flow photoreactors are likely better suited for mediating reactions with hazardous chemicals or reactions involving gaseous reagents than a mechanophotocatalysis approach.[84] On the other hand, mechanophotocatalysis should be used to mediate heterogeneous reactions that are difficult to adapt to flow reactors, thus enabling complementary use cases between these two technologies.’

2) Additionally, referencing the recent review J. Am. Chem. Soc. 2025, 147, 32, 28523–28545 would be valuable, as it provides a topical complement to this Outlook and situates the discussion within the latest literature.

Our response: We thank the reviewer for this suggestion. We have cited the review when we first discuss the development of new enabling reactor technologies on page 12:

‘We also anticipate the development of new enabling reactor technologies[84] that facilitate high-throughput screening of reactions and reaction scale-up.’

We have also cited this work in the paragraph discussing the limitations of mechanophotocatalysis, when discussing situations where flow chemistry in solution may be preferable.

Overall, this is a thoughtful, well-written, and forward-looking contribution that is likely to attract attention and stimulate further research in this exciting emerging field. It provides a clear and accessible roadmap for both chemists entering the area and those seeking to understand how mechanophotocatalysis may reshape sustainable photochemical synthesis in the years ahead.

Reviewer: 4

Recommendation: Publish in ACS Central Science after minor revisions noted.

Comments:
The manuscript proposed as an Outlook article by Millward and Zysman-Colman is focused on recent developments and prospects of mechanophotocatalysis as an emerging methodology within the realm of solid state mechanochemistry. The authors have listed important publications in the field in a chronological order, showing how photocatalysis in the solid state has evolved from the first simple rod mill-type setup to vibrational ball milling and resonant acoustic mixing, as well as the increase in complexity of organic transformations carried out under these conditions. As a relatively new addition to the mechanochemical toolbox, mechanophotocatalysis is shown as a promising alternative to solution-based photocatalytic reactions, and disscussed in terms of potential niches for future investigations. As such, it is expected that research in mechanophotocatalysis will gain momentum as our understanding of symbiosis of milling and photochemistry broadens in the years to come. The manuscript itself is well written and is suitable for publication in ACS Central Science after minor revision:

Our response: We thank the reviewer for their positive assessment of the outlook, and we will address each of their suggestions here.

1. Page 2, line 25: "... due to its ability to mediate solvent-minimized versions of known solution-state reactions". Not only this, mechanochemistry allows access to organic molecules not stable in solution environment (reactive intermediates) as well as the discovery of solid-state reactivity different from the one observed in solvents. Refs might include Angew. Chem. Int. Ed. 2015, 54, 8440. and Angew. Chem. Int. Ed. 2014, 53, 9321.

Our response: The reviewer makes an excellent point here. We have added the suggested references and modified the text on page 2 to the following:

Aside from reducing waste generation associated with reaction solvent,[41] mechanochemical reactions can be faster,[27, 42] can facilitate access to new chemical species and reaction selectivities,[43, 44] and be more tolerant to aerobic conditions[39, 45] and poorly soluble reaction components[42, 46-48] than their solution-state analogues, enabling simplified workflows and access to new chemical space.

We feel that the inclusion of these benefits of mechanochemistry is more appropriately placed here, than at the start of the section as suggested by the reviewer.

2. Page 2, lines 26, 28 and Figure 1: I'm not sure that the term "transmutation" is the most appropriate one to describe the use of mechanochemistry in place of conventional solution-based approach.

Our response: We had used this term in our previous work (*Angew. Chem. Int. Ed.* **2024**, *63*, e202316169 and *J. Am. Chem. Soc.* **2025**, *147*, 22919-22931) as we liked how it conveyed the idea of 'changing something from one state or form to another'. However, we appreciate that other words may be more appropriate for conveying the translation of reactions from the solution state to a new environment. We have thus removed the term from the perspective, and in the figure, we have used the term 'mechanochemistry adaptation', as this more appropriately conveys the idea of moving a reaction to a new environment.

3. Page 4, line 5: "...while others have used the terms solid-state photochemistry,49,50 or photomechanochemistry.51-53"

For the sake of accuracy, the term "solid-state photochemistry" is used in ref 49 and "mechanochemically-assisted solid-state photocatalysis" in ref 50. Please refer to this as well.

Our response: We apologise for this oversight; the issue has now been corrected.

4. Page 4, line 44: Besides chemical resistivity, the opacity of PMMA jars represents another obstacle for an efficient photochemical reaction to proceed.

Our response: This is a good point, and we have now mentioned this in the text:

> 'While transparent milling jars made from polymethylmethacrylate (PMMA) are used in mechanochemistry

applications, particularly for *in situ* reaction monitoring,[69] they frequently lack the required chemical resistivity to be useful for the majority of photocatalysis reactions, and can become scratched and opaque over time, leading to poor light penetration.[54]'

5. Page 5, Figure 2. In my opinion, the figure would probably work better if it was split into several figures, each placed in the respective paragraphs. In this way, it would be much easier for the reader to follow the text and see the transformations discussed. Please take into consideration.

Our response: This is an excellent suggestion, which was also made by Reviewer 1. We agree that this Figure is too crowded; we had hoped to concisely show all the current examples in one place as a reference for readers, but we recognise that in its current form the figure could be overwhelming for the reader. To address this issue, we have converted this figure into three separate single column figures:

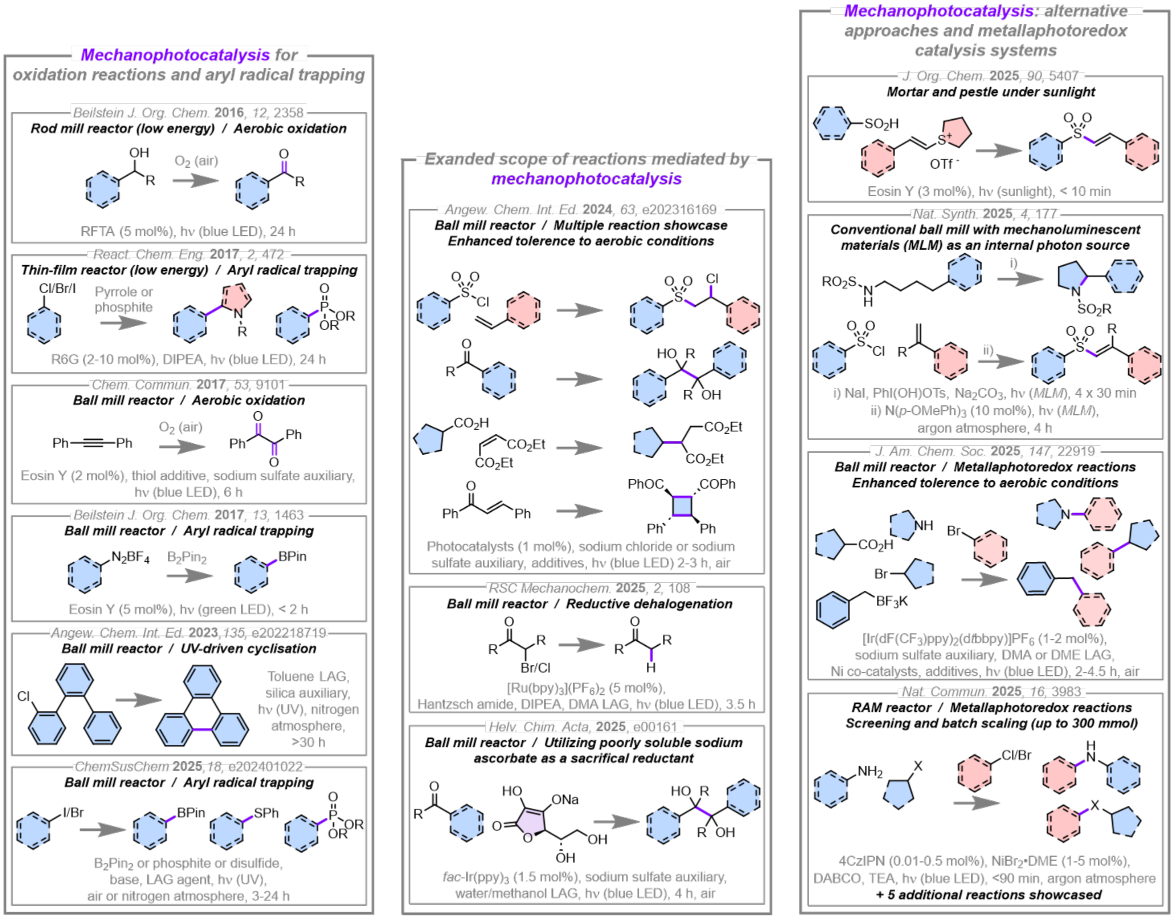

6. Page 6, line 26: I suggest the sentence "In 2017,..." starts with a new paragraph.

Our response: We agree that this is a necessary break in the text, and we have made this change.

7. Page 6, lines 29 and 32: The reactions described in refs 50 and 62 were photocatalytic and used Eosin Y as a photocatalyst, this should be added. Also, ref 50 reported on the use of LED strips for merging photocatalysis and ball milling, as well as on the design of a LED photoreactor compatible with simultaneous ball milling. In ref 62, LED strip was

employed. These two approaches have later been adopted (with minor modifications) by other research groups and thus represent an important contribution to the field of mechanophotocatalysis. Therefore I suggest that a scheme is inserted here showing these two designs, as they are easy to implement and readers may find it useful for their research.

Our response: We thank the reviewer for their suggestions. We have updated the text to include the explicit mention of Eosin Y as a photocatalyst for these references. We have also now made explicit mentions of how light irradiation was achieved in each example. We have chosen to discuss this in the text, rather than in the figures, as we are trying to showcase the reactions that have been developed with more emphasis, while a previous review has focused more in depth upon showing images of many of these reactor designs (Beilstein J. Org. Chem. 2025, 21, 458).

8. Page 7, line 25: In relation to my previous comment, it would be also nice to see the reaction vessel design from the author's group. This could be included in the same scheme with LED reactor/strip or as a separate scheme.

Our response: We thank the reviewer for their suggestion. In line with our previous comment, we wanted this outlook to be broadly forward looking, and have thus focused the schemes on the types of reactions that have been explored. We have thus opted not to show any specific reactor designs in this outlook.

9. Page 8, line 34: What is 4CzIPN? Please provide a full chemical name.

Our response: We have added the full name of 4CzIPN (1,2,3,5-tetrakis(carbazol-9-yl)-4,6-dicyanobenzene) to the text and the Figure caption.

10. Page 9, line 16: RAM reaction scale up - what is the typical scale of these reactions in RAM?

Our response: We have added an additional clarification, using the work of Rueping and co-workers as an example:

‘We also anticipate the development of new enabling reactor technologies[84] that facilitate high-throughput screening of reactions and reaction scale-up. In this context, RAM reactors have shown particular promise, as these platforms can be used for both

applications in the same reactor (for example, Rueping and co-workers could conduct reactions at a range of scales from 0.4-300 mmol).[60]’

11. Page 10, line 23: "a" should be deleted.

Our response: We thank the reviewer for noticing this error, it has now been sorted.

12. Page 10, line 26: A reference for large-scale industrial photocatalytic processes should be placed here.

Our response: We have added a review containing an overview of various scale-up examples of photocatalytic reactions.

13. Page 11, Figure 3. I suggest to add "scale up" under Technology innovations, as an important aspect of full implementation of mechanophotocatalysis in the future.

Our response: This is an excellent suggestion; we have added this to the figure.

I thank you for your time in handling our manuscript.

Sincerely,

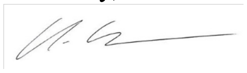

Eli Zysman-Colman on behalf of the co-authors
